# Characteristic distribution of eicosapentaenoic acid in human abdominal aortic aneurysm wall

**DOI:** 10.1016/j.jlr.2022.100200

**Published:** 2022-03-18

**Authors:** Hirona Kugo, Hiroki Tanaka, Tatsuya Moriyama, Nobuhiro Zaima

**Affiliations:** 1 Department of Applied Biological Chemistry, Graduate School of Agriculture, Kindai University, Nara, Japan; 2 Department of Medical Physiology, Hamamatsu University School of Medicine, Hamamatsu, Japan; 3 Agricultural Technology and Innovation Research Institute, Kindai University, Nara, Japan

**Figure:**
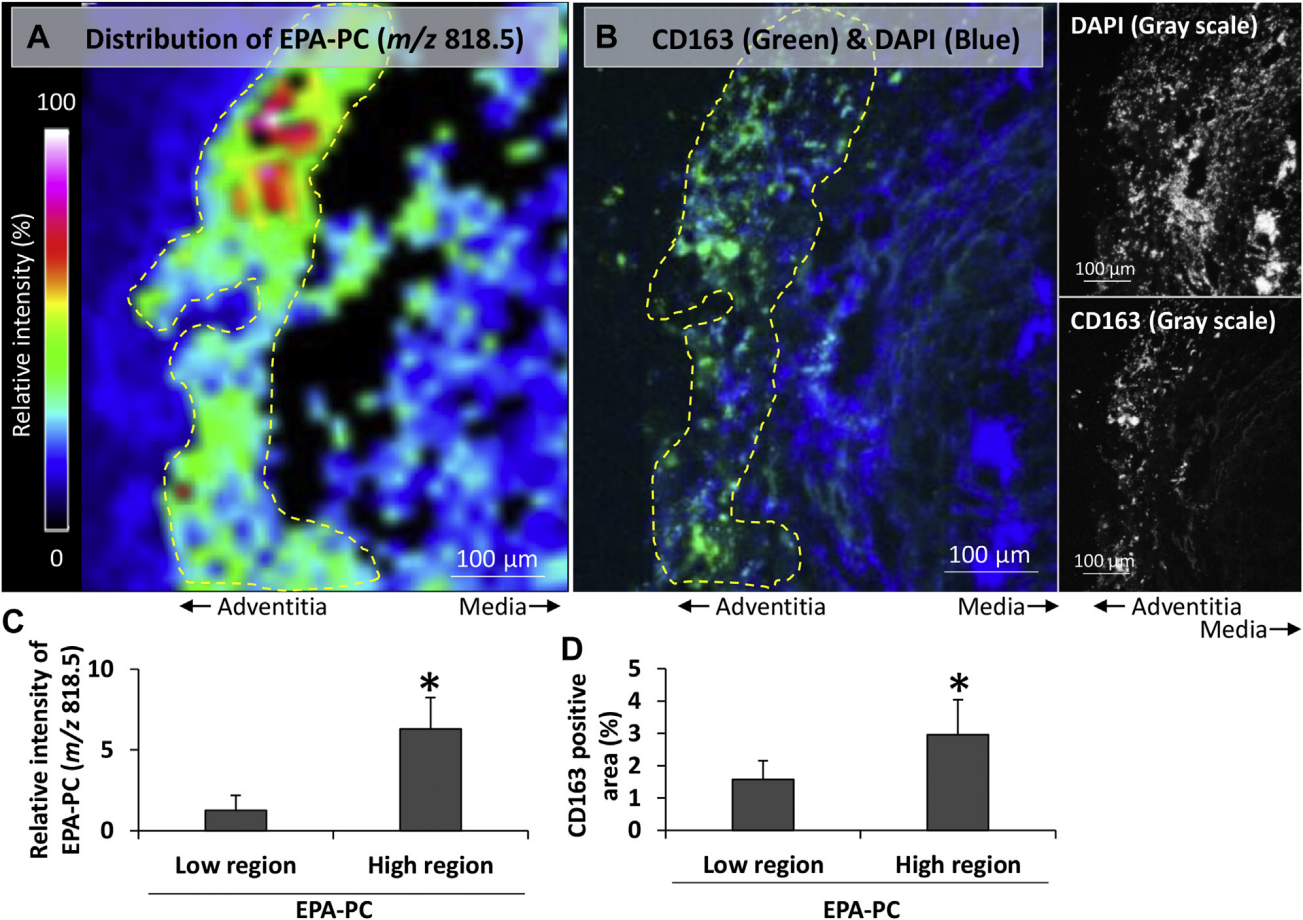

Abdominal aortic aneurysm (AAA) is a vascular disease in which the abdominal aorta gradually dilates. Dilation of AAA is associated with the aortic wall weakness induced by chronic inflammation (1). Several epidemiologic studies reported that consumption of fish is associated with the prevention of AAA. Animal and human studies suggested n-3 PUFAs can protect against AAA development and rupture. These previous data provide evidence to suggest the suppressive effect of n-3 PUFAs on AAA, which has no pharmaceuticals; however, the distribution of n-3 PUFAs in the aorta is a “missing link” to understand mechanisms underlying suppressive effects of them. Here, we visualized the distribution of EPA-containing phosphatidylcholine (EPA-PC) (*m/z* 818.5) in the human AAA wall by MALDI-mass spectrometry imaging (MALDI-MSI), which can visualize the distribution of molecules in tissue sections (2). Postoperative human AAA tissues were collected after obtaining informed consent. This study was approved by the Ethical Review Committee of the Hamamatsu University School of Medicine (approval number: E15-139). Fresh frozen tissues were used for MALDI-MSI. MALDI-MSI was performed as described previously (3). EPA-PC was not ubiquitously distributed in the AAA wall (A, yellow dotted line). The distribution of EPA-PC was similar to that of M2 macrophage marker (CD163) in an adjacent section (B, yellow dotted line). When the regions in the AAA wall were divided into two groups (EPA-PC-low and EPA-PC-high regions), intensity of EPA-PC in the EPA-PC-high region was six times higher than that in the EPA-PC-low region (C), and M2 macrophage marker-positive area in the EPA-PC-high region was significantly higher than that in the EPA-PC-low region (D). Similarity between the distribution of EPA-PC and M1 macrophage marker was not observed. These observations are consistent with our previous study that reported the similar distribution of EPA-PC and M2 macrophages in the AAA wall of experimental model animals, which were administered EPA-rich fish oil (3). While M1 macrophages have proinflammatory function, M2 macrophages have anti-inflammatory function (4). The anti-inflammatory function of M2 macrophages is attributed to the production of anti-inflammatory eicosanoids, which are produced from EPA. Characteristic distribution of EPA in the human AAA wall could result from the different requirement for fatty acids of cells in the AAA wall and provide new clues to understand the suppressive effect of n-3 PUFA on AAA.

**EQUIPMENT:** Autoflex Speed KN2 (Bruker Daltonics).

## Conflict of interest

The authors declare that they have no conflicts of interest with the contents of this article.
